# Technology Used to Recognize Activities of Daily Living in Community-Dwelling Older Adults

**DOI:** 10.3390/ijerph18010163

**Published:** 2020-12-28

**Authors:** Nicola Camp, Martin Lewis, Kirsty Hunter, Julie Johnston, Massimiliano Zecca, Alessandro Di Nuovo, Daniele Magistro

**Affiliations:** 1 Department of Sport Science, School of Science and Technology, Nottingham Trent University, Nottingham NG11 8NS, UK; nicola.camp@ntu.ac.uk (N.C.); kirsty.hunter@ntu.ac.uk (K.H.); Julie.johnston@ntu.ac.uk (J.J.); 2 Department of Sport and Exercise Science, University of Derby, Derby DE22 1GB, UK; M.Lewis@derby.ac.uk; 3 Wolfson School of Mechanical, Electrical and Manufacturing Engineering, Loughborough University, Loughborough LE11 3TU, UK; m.zecca@lboro.ac.uk; 4 Department of Computing, Sheffield Hallam University, Sheffield S1 1WB, UK; a.dinuovo@shu.ac.uk

**Keywords:** wearable technology, environmental sensors, autonomous living

## Abstract

The use of technology has been suggested as a means of allowing continued autonomous living for older adults, while reducing the burden on caregivers and aiding decision-making relating to healthcare. However, more clarity is needed relating to the Activities of Daily Living (ADL) recognised, and the types of technology included within current monitoring approaches. This review aims to identify these differences and highlight the current gaps in these systems. A scoping review was conducted in accordance with PRISMA-ScR, drawing on PubMed, Scopus, and Google Scholar. Articles and commercially available systems were selected if they focused on ADL recognition of older adults within their home environment. Thirty-nine ADL recognition systems were identified, nine of which were commercially available. One system incorporated environmental and wearable technology, two used only wearable technology, and 34 used only environmental technologies. Overall, 14 ADL were identified but there was variation in the specific ADL recognised by each system. Although the use of technology to monitor ADL of older adults is becoming more prevalent, there is a large variation in the ADL recognised, how ADL are defined, and the types of technology used within monitoring systems. Key stakeholders, such as older adults and healthcare workers, should be consulted in future work to ensure that future developments are functional and useable.

## 1. Introduction

Population ageing affects many countries globally and is associated with substantial social, financial, and health challenges [1]. In the UK, for example, it is predicted that a quarter of the population will be aged 65 or over by 2050 [2]. The ageing process is characterised by a gradual loss of physical and cognitive abilities; thus, the maintenance of functional independence over time has been, and remains, one of the most important goals pursued by healthcare systems. However, these systems are often poorly equipped to provide effective, age-appropriate care for those with chronic conditions [3], particularly due to an increasing number of people requiring care compared to those able to provide it [4].

To overcome these issues, current trends in geriatric care are increasingly allowing or assisting older people to live independently in their own homes [3,4]. This concept, known as “ageing-in-place”, is thought to positively contribute to an individual’s overall well-being and healthy ageing, while reducing the cost of healthcare associated with moving older adults into supported living environments [5,6]. However, the current adaptations in healthcare to support this concept are often inefficient and fragmented [6], which places an increased burden on family members or other informal caregivers to provide support [4]. To limit the impact on informal caregivers, and to ensure that community-based care is appropriate for an individual, assessment is made of the older adults’ physical or perceived ability to undertake Activities of Daily Living (ADL) [7].

Activities of Daily Living (ADL) are the “fundamental activities needed for an individual to function independently in everyday life” [8]. Typically, these are condensed into six basic ADL (BADL): bathing, feeding, toileting, transferring, mobility, and continence [9]. However, there are inconsistencies relating to the activities that should be included, particularly when referring to individuals living independently in a community setting. It can be argued that more complex instrumental ADL (IADL), such as managing money and medication, housekeeping, and using a telephone, are equally important [10,11]. However, a recent review found that IADL are either not included in current, performance-based tests or are only superficially considered [12]. Furthermore, activities relating to socializing and communication (SADL) are often ignored despite the relationship between social engagement and functional decline [13,14].

The scales used to measure ADL function vary in terms of the type of ADL measured, the specific activity, and the level of detail assessed [15]. This may be due to their development, which has typically been through adapting previous versions, rather than based on any consistent framework or theory [16]. To counteract these inconsistencies, performance testing through observation is often used [10]. However, observations are also subjective measures, and therefore may not measure a person’s true abilities, particularly because they are typically made within a clinical setting that cannot reflect adaptations made in a person’s everyday living situation [12,17,18]. For example, a firm, higher chair (such as those found in hospitals) is arguably easier to stand up from than a low, softer chair (such as a well-used sofa, which typically exist in an older person’s home). Therefore, everyday functioning is not being accurately assessed, and the individual may be classified as more able than they are. As a result, interventions or assistance may not be provided at the most appropriate time or at a suitable level for the individual, reducing their effectiveness and leading to the older adult experiencing greater functional decline and a greater risk of nursing home admission [7].

To overcome these issues, it has been suggested that objective measures performed within a home environment may be the most appropriate method of assessing functional ability [10,17,19,20]. Objective measures are not influenced by the practitioner or the subject in the same way as subjective measures as they are not reliant on perceptions of performance [21]. Through the removal of a human element, and introduction of a technological component, objective measures can provide a more reliable measure of functional ability [19]. Technological devices such as wearable accelerometers are less influenced by external factors, with measurements dependent on the design of the device and the algorithm used; whatever error is present will be consistent each time the device is used and can therefore be accounted for. However, traditional measures often separate the physical and cognitive elements of ADL function, despite the clear correlations between the two elements [7,22], and therefore there is a clear need to develop methods of ADL recognition and measurement that incorporate both elements.

Typically, objective ADL measurements are collected from wearable sensors, such as accelerometers; environmental sensors, such as motion detectors; or other technology, such as cameras [20]. Data from these types of technology can be combined, creating ubiquitous monitoring “systems” [21]. These systems are capable of recognising a variety of ADL, such as stair climbing, meal preparation and cleaning [23,24], as well as an individual’s ability to acknowledge that a task needs to be completed [22]. These monitoring systems are not designed to replace traditional measures, but rather to aid healthcare workers in identifying potential declines, which can then be specifically assessed. A monitoring system may be able to highlight which specific ADL an individual is struggling with, allowing a more targeted assessment to take place rather than assessing all ADL each time, some of which may be irrelevant for the individual [25].

Ubiquitous monitoring systems are capable of continuous monitoring, something that traditionally would only be achieved by having a caregiver constantly present, which is both impractical and may lead to changes in an individuals’ performance [26]. The continuous monitoring aspect of these systems also allows them to “learn” normal behaviour patterns, and therefore recognise significant deviations in performance immediately without the need for specific testing. They would also be able to detect the more subtle changes in function associated with early functional decline, which has been highlighted as a significant challenge that current measures are unable to identify [27]. Caregivers can subsequently be alerted to potential functional decline, allowing appropriate interventions to be put in place before the situation becomes critical [28]. Another potential benefit of continuous monitoring is the ability to monitor an individual’s capability to self-initiate a task when necessary. This has been described as a key issue in dementia and MCI [18] but can currently only be assessed if a caregiver is present at the time. A continuous monitoring system would allow self-initiation to be recognized, as well as the physical ability to perform a given ADL, providing a more complete analysis of an individual’s functional ability. Currently, monitoring systems are not able to replace the traditional measures due to several factors, such as the acceptance of technology by older adults and healthcare professionals, the ADL recognised (and the level of detail included), and the technology used. However, there is great potential for these systems as an aid for decision-making processes among healthcare staff relating to interventions for community-dwelling older adults. This could subsequently help to reduce caregiver burden and overall healthcare cost relating to institutionalization and other healthcare needs [4,26,29].

This scoping review aims to investigate the current technology that is capable of recognizing the activities of daily living of older adults in a free-living environment. It includes technological monitoring systems that have been implemented in the homes of older adults, including commercially available systems and those that have been used in research only. This review will also explore how technological monitoring systems can aid healthcare by comparing their capabilities with existing ADL measures, highlighting how the two approaches can be used together. Specifically, it aims to answer the following questions:

(1) Which ADL are recognised by technology?
(2) What types of technology are currently used to recognise ADL in free-living older adults?
(3) How are ADL recognised by technology (inferred/direct measurement)?

As the area of objectively monitoring ADL function is developing rapidly, with many new advances being disseminated through conference papers and commercial systems being released without being published in academic papers, a scoping review was deemed the most appropriate approach, as it allows for the inclusion of “grey literature” [30]. There is a danger, if these separate areas are not synthesised, that developers and researchers will be focusing on the same things and wasting time and resources. This scoping review therefore also aims to bring all recent advances together and highlight where the true gaps currently are, ensuring that future research projects are both relevant and required.

## 2. Materials and Methods

**Figure 1:**
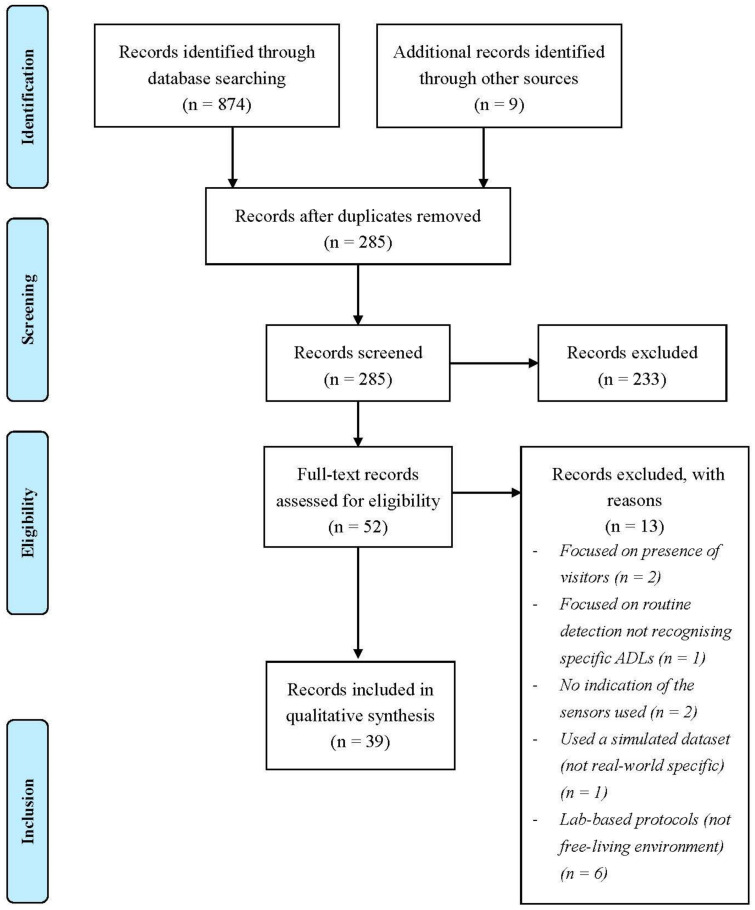
PRISMA paper selection flowchart, adapted from [32].

The aim of this review was to identify the technology capable of monitoring ADL function of independently living older adults without cognitive impairment. A scoping review was conducted following the Preferred Reporting Items for Systematic Reviews and Meta-Analyses extension for Scoping Reviews (PRISMA-ScR) guidelines [31]. The search strategy included a systematic search of the existing literature, and a Google search to identify existing commercial systems (Figure 1). The searches were carried out between January and August 2020.

Reference [33] summarised the research relating to home-monitoring of older adults, therefore only published research from 2008 onwards was included. Commercial systems were included if they are available currently regardless of the year they were introduced. Other inclusion and exclusion criteria are summarised in Table 1.

The search terms used (Table 2) were based on previous reviews related to the use of technology to recognise the ADL of older adults [33,34,35,36,37]. Search terms were divided into four property groups—population, activity, measurement, and technology. To be included in the paper selection process, the title or abstract had to contain at least one key term from at least three of the property groups [38]. The literature search was conducted within the publication databases of PubMed, Scopus, and Google Scholar, using the Boolean operator “OR” within the property groups and “AND” between the property groups.

## 3. Results

This review had three main objectives: (1) identify the ADL that are recognised; (2) identify the types of technology currently used; and (3) identify whether the ADL are recognised from direct measures or inferred from sensor data. The search process identified 14 ADL, 20 types of technology, and 39 systems, separated into three categories (environment only, wearable only, and environmental + wearable).

Currently available systems recognise a mixture of BADL, IADL, and SADL (Table 3). Most of these are present in current subjective measures, such as the Barthel Index or Lawton and Brody scale; however some are not traditionally included in the assessment of independent living ability, namely, “sleep”, “bed use”, “stair use”, and “social interaction”. “Feeding”, “grooming”, and “household” have been used as umbrella terms for combinations of sub-activities, at least one of which had to be recognised by the system to be included. For example, “feeding” includes systems that recognised eating activity only, meal preparation activity only, and both types of activity.

The details of each identified system, including the sensors used, ADL recognised, and outcome measures, are provided in Supplementary Material (see Table S1) and have been assigned a number that will be referred to throughout the rest of this review. Thirty-nine systems were identified, separated into four types: commercially available but not used in research, commercially available and used in research, named sensors, and unnamed sensors. Only two of the nine commercially available systems have been used in research, one of which was used in conjunction with other types of sensor. Seventeen of the systems identified did not name the specific sensors used.

ADL monitoring systems can be arranged into three categories (Table 4):

- Environmental only (*n* = 36): systems using only environmentally based technology, such as motion sensors and video cameras [10,19,20,23,24,25,26,28,37,40,41,43,44,45,46,47,48,49,50,51,52,53,54,55,56,57,58,59,60,61,62,63,64,65,66,67,68].
- Wearable only (*n* = 2): systems using only body-worn technology, such as accelerometers [39,51].
- Environmental + wearable (*n* = 1): systems using a combination of body-worn and environmentally based technology [39].

Most systems are environmental only, with the most common combination being door + motion, especially in recent years. Wearable technology is rarely included, being present in just three of the 39 systems. One of these specifically focused on “feeding”; however, it did recognise specific activities within this, such as drinking and using cutlery, which was not included in other systems [69]. On the other hand, the other systems, which included wearable technology, were used to recognise a large number of ADL compared to many of the environmental only systems, suggesting that they may provide a greater level of detail relating to movements than what the other systems are capable of.

Within each system category, varying combinations of technology types are used to create the separate systems. Where there are multiple references for each sensor combination, for example the combination of door + motion [41,43,45,48,61,64], the separate systems have been used to recognise different ADL.

**Table 4:** Summary of the identified systems, including the system category, technology type included, and the ADL recognised. The systems corresponding to the references can be found in Table S1.

System Category	Technology Combination	Reference	Year	Total Number of ADL Recognised	Bed Usage	Dressing	Feeding	Grooming	Household	Medicine	Mobility	Recreation	Sleep	Social Interaction	Stair Usage	Toileting	Transferring	TV Usage
Environmental Only	Depth camera	[53,54]		5			✓		✓		✓	✓						✓
		[53]	2014	3			✓				✓							✓
		[54]	2015	4			✓		✓		✓	✓						
	RFID Tag	[24]	2012	8	✓	✓	✓	✓	✓			✓				✓		✓
	Motion	[10,55,56,57,58]		11	✓	✓	✓	✓			✓	✓	✓	✓		✓	✓	✓
		[55]	2008	6	✓	✓	✓	✓								✓	✓	
		[56]	2008	5			✓	✓				✓	✓			✓		
		[57]	2010	6			✓	✓				✓	✓	✓				✓
		[58]	2014	2				✓								✓		
		[10]	2018	7			✓	✓			✓	✓	✓	✓		✓		
	Power consumption	[51]	2011	4			✓	✓	✓							✓		
	Video Camera	[23]	2016	8	✓		✓	✓				✓	✓	✓		✓		✓
	Door + Motion	[41,43,45,48,61,64]		11	✓		✓	✓	✓	✓	✓	✓	✓	✓		✓	✓	
		[45]	2004	3				✓			✓			✓				
		[61]	2015	9	✓		✓	✓	✓	✓		✓	✓	✓			✓	
		[48]	2017	3				✓			✓			✓				
		[64]	2017	4			✓					✓	✓	✓				
		[43]	2018	4							✓		✓	✓		✓		
		[41]	2019	6			✓	✓			✓		✓	✓		✓		
	Force/pressure + Power consumption	[52]	2013	6	✓		✓	✓				✓				✓		✓
	Hydro + Motion	[28]	2016	4				✓	✓		✓					✓		
	Motion + Power Consumption	[37,66]		7			✓	✓	✓			✓	✓	✓				✓
		[37]	2012	4			✓	✓					✓					✓
		[66]	2019	6			✓	✓	✓			✓	✓	✓				
	Door + Motion + Power consumption	[47,50,67]		5			✓	✓			✓			✓				✓
		[47] ^a^	2015	5			✓	✓			✓			✓				✓
		[50] ^a^	2019	5			✓	✓			✓			✓				✓
		[67]	2020	5			✓	✓				✓	✓	✓				
	Door + Motion + Video Camera	[44]	Unknown	4			✓	✓			✓			✓				
	Door + Light + Motion + Temperature	[46,63]		7			✓	✓			✓	✓	✓	✓			✓	
		[46]	2013	3				✓			✓			✓				
		[63]	2016	6			✓	✓				✓	✓	✓			✓	
	Door + Motion + Power consumption + Temperature	[25]	2014	3		✓	✓	✓										
	Force / Pressure + Motion + Power consumption + Temperature	[59,65]		6			✓	✓				✓	✓			✓		✓
		[59]	2014	5			✓	✓				✓	✓			✓		
		[65]	2018	6			✓	✓				✓	✓			✓		✓
	Accelerometer + Door + Humidity + Light + Motion	[26]	2011	8	✓		✓	✓		✓		✓	✓	✓			✓	
	Accelerometer + Humidity + Light + Motion + Temperature	[20,40]	2014/15	7	✓		✓	✓				✓	✓			✓		✓
	Door + Light + Motion + Sound + Temperature	[49]	2018	4			✓	✓			✓			✓				
	Accelerometer + Door + Humidity + Motion + Power consumption + Temperature	[62]	2015	6	✓	✓	✓	✓			✓		✓					
	Door + Grid-eye + Light + Motion + Power consumption + Temperature	[19]	2019	5			✓	✓					✓	✓		✓		
	Accelerometer + Door + Humidity + Motion + Power consumption + Sound + Temperature	[60]	2014	5		✓	✓	✓			✓						✓	
	Barometer + Door + Humidity + Light + Motion + Sound + Temperature	[68]	2020	4			✓	✓					✓	✓				
Wearable Only	Accelerator + Gyroscope	[69]	2019	1			✓											
	Accelerometer + Altimeter + Barometer + Gyroscope + Light + Temperature	[42]	2015	8			✓	✓	✓		✓	✓	✓		✓			✓
Environmental and Wearable	Accelerometer + Motion	[39]	2012	6		✓	✓				✓	✓	✓			✓		

^a^ Although these systems used the same technology to recognise the same ADL, they are different commercially available systems and therefore differ in terms of the specific properties of the technology and are therefore separately included.

### 3.1. Recognised ADL

**Figure 2:**
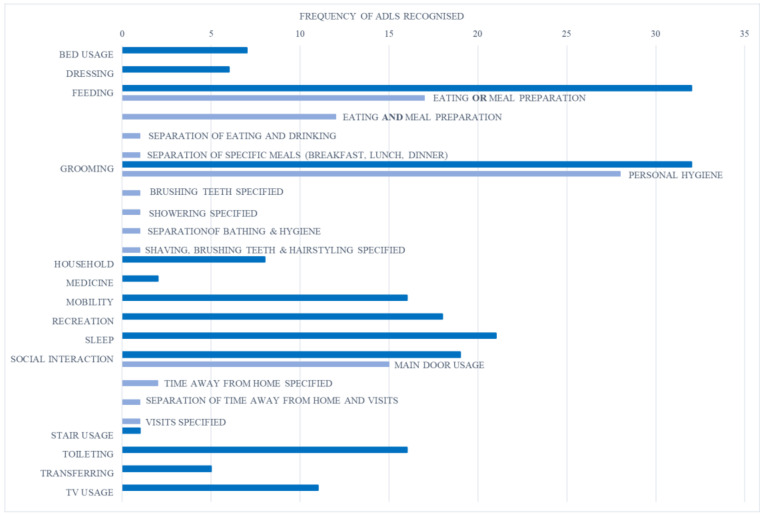
Frequency of ADL recognition by the identified systems. “FEEDING”, “GROOMING”, and “SOCIAL INTERACTION” include sub-activities, which were specified by some systems.

No single system recognised all 14 of the identified ADL (Figure 2). Feeding and grooming were the most frequently recognised ADL, being recognised by 33 and 32 of the systems, respectively. Of the 33 systems recognising “feeding”, 18 included either eating or meal preparation [25,37,39,42,44,47,49,50,53,54,55,56,57,60,64,65,66,67]; 12 included eating and cooking as separate activities [10,20,23,24,26,40,41,51,52,59,61,63,68]; one distinguished eating from drinking [69]; and one recognised the preparation of breakfast, lunch, and dinner as separate ADL [19]. Most systems that recognised “grooming” defined these ADL as “personal hygiene activities”; however, [42] specified “brushing teeth” only, [23] specified “showering” only, [26] separated “bathing” from “hygiene”, and [20/40] divided these ADL into four separate ADL: “showering”, “shaving”, “brushing teeth”, and “styling”. “Social interaction” referred mainly to front door usage; however, [23] and [64] specified “time away from home”, [43] separated “visitors” from “time away from home”, and [57] specified “visitors”. The least recognised ADL were “medicine”, recognised by two systems [26,61], and “stair usage”, recognised by just one system [42].

### 3.2. Identified Technology Types and Recognition Systems

**Figure 3:**
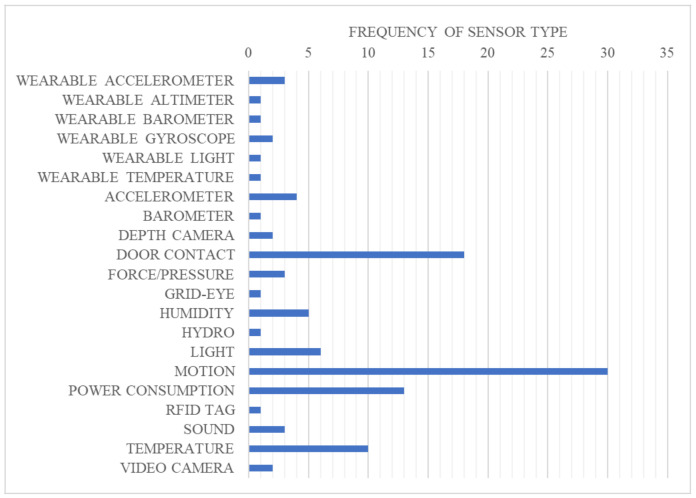
Frequency of sensor types used in the identified systems. They are environmental sensors (placed around the home) unless stated otherwise.

The search identified 21 types of technology, including six wearable sensors, 13 environmental sensors, and two types of camera (Figure 3). Motion sensors were the most common, present in 30 of the systems, followed by door contact and power consumption (Figure 3). Grid-eye [19], hydro sensors [28], and RFID tags [24] were among the least common, appearing in only one system each (Figure 3). Wearable sensors were rarely used, present in only six of the 21 identified sensor types. Within this, three systems used a wearable accelerometer [39,42,69], two used a wearable gyroscope [42,69], and only one system included a wearable altimeter, wearable barometer, wearable light sensor, and wearable temperature sensor [42] (Figure 3).

**Figure 4:**
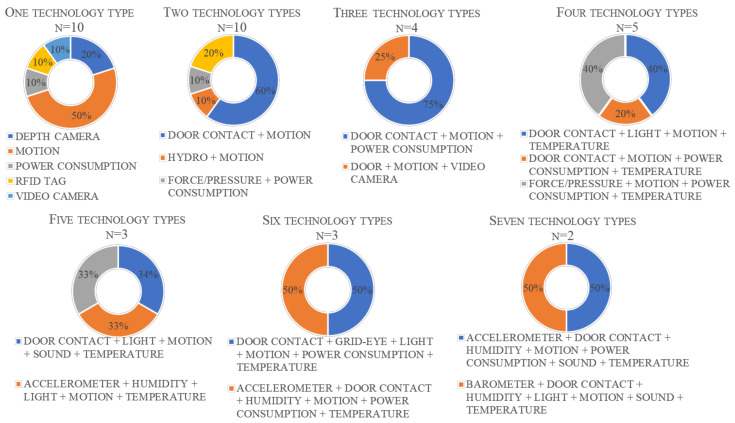
Combination of technology types used within the environment only system category; *n* denotes the number of systems using the specified number of technology types, with the technology combinations shown below each chart.

Figure 4 visualizes the environment-only category due to the large variation of technology combinations within this category. Most of the environment-only category systems used either one type of technology (*n* = 10) [10,23,24,51,53,54,56,57,58] or a combination of two types of technology (*n* = 10) [37,41,43,45,48,52,61,64,66,69]. Of the 24 systems using a combination of two or more types of technology, only one did not include motion sensors [52] and only two systems used a combination of seven technology types [60,68].

Two systems were categorized as wearable only (Table 4), one utilizing just a wearable accelerometer and wearable gyroscope [69] and one using a combination of a wearable accelerometer, wearable altimeter, wearable barometer, wearable gyroscope, wearable light sensor, and wearable temperature sensor [42]. Only one system was categorized as an environmental + wearable system (Table 4), consisting of a wearable accelerometer and motion sensor [39].

Interestingly, the systems with the most sensor types [60,68] did not recognise the most ADL; only recognizing 5 and 4 of the 14 identified ADL, respectively. The largest number of different ADL were recognised by the systems using only motion sensors [10,55,56,57,58], and the systems using a combination of door + motion sensors [41,43,45,48,61,64], which were each used to recognise 11 of the 14 identified ADL (Table 4). In contrast, [69] only recognised one ADL—feeding—which was the fewest of any system. However, it should be acknowledged that this system recognised several smaller activities within the ADL classification of “feeding”, such as separating eating and drinking activity.

### 3.3. ADL Recognition Method (Direct/Indirect)

The identified systems use either direct measurement, indirect inference, or a combination of both methods to recognise ADL (Table 5). Directly measured activities are those that are recognised from sensor data that cannot be mistaken for another activity. For example, “TV usage” is often recognised from power consumption sensors connected to a television: if power is detected by this specific sensor, the television must be on. In contrast, indirect measures infer that an activity is likely to have taken place but does not have the same certainty level offered by the direct measure. For example, “dressing” can be inferred via data from door sensors attached to a wardrobe, but the activity being measured is the opening of the door and not the action of dressing. The fact that a door has been opened does not necessarily mean that an individual is physically able to dress themselves, but that they are aware of the need to change clothes. The same is true of “medicine”; the act of opening a medicine cabinet does not show that the medicine has been taken but does show that an individual is aware that medicine is needed.

Of the 14 ADL, nine were recognised indirectly, meaning that they were inferred from sensor data such as room movement or water consumption (Table 5). Only two activities—bed usage and TV usage—are measured directly by current systems and the remaining three are recognised using both direct and indirect methods (depending on the system). Often it is the combination of different sensor information that allows the system to recognise an activity, rather than a single sensor. For example, “grooming” includes bathing, which can be inferred through water usage combined with bathroom activity, although the individual components relating to washing are not assessed. The three activities recognised through both direct and indirect methods are commonly the result of wearable sensors alone or combined with motion sensors. This allows the room a movement occurs in to provide context to the activity and therefore the recognition to be more accurate.

## 4. Discussion

This scoping review aimed to identify the types of technology currently used to recognise the activities of daily living of older adults within a free-living environment. The use of technology has been suggested as a means of allowing continued autonomous living while providing support and monitoring functional ability, allowing early interventions where needed [21]. Three key areas relating to ADL recognition systems were identified: the activities recognised, common technology types included, and the recognition method used (direct or indirect).

### 4.1. ADL Recognised and Compared to Usual Scales

Overall, the systems identified through this review recognised 14 different ADL, ranging from BADL, such as feeding and grooming; IADL, such as medicine and household; and SADL, associated with social interaction (Figure 4).

It should be noted that the systems identified are designed to monitor whether an ADL action has been completed, rather than testing the individual’s ability to complete an ADL action. However, there is an overlap between the two approaches, which if capitalized on effectively could make healthcare more targeted and therefore more efficient. For example, seven of the recognised ADL (bathing, dressing, feeding, grooming, mobility, stairs, toileting, and transfer) are also present in the common assessment scale of the Barthel Index [70]. However, the sub-activities associated with these ADL differ. For example, “feeding” in the Barthel Index relates to the ability to carry out specific, fine skills, such as spreading butter or cutting food [71], whereas technology-based systems can recognize sub-activities related to environmental interaction, such as opening cupboards and using heating devices. If an individual starts to show signs of decline related to “feeding”, i.e., the monitoring systems detects a difference in the movements or a complete lack of “feeding” activity, a healthcare professional can then carry out targeted testing related to that specific ADL to identify what the issue is and provide a precise intervention. In essence, the monitoring systems allow testing to be more specified and relevant to the ADL that an individual are struggling with, rather than carrying out a battery of tests that may be irrelevant for the individual, thereby making healthcare more efficient.

Although the ability to recognise several sub-activities is undeniably helpful in identifying where functional decline may be occurring, there needs to be a consensus on which sub-activities are genuinely helpful to monitor compared to the ones being recognised purely because the technology is capable. For example, inferring “feeding” from kitchen activity and object interaction, compared to identifying specific movements, such as spreading butter and using cutlery [19]. It has been well established that the current aims of these systems are to enhance the effectiveness of care relating to the decision-making about a person’s needs; however, few recognise fine skills such as cutlery use, which can have a large influence on more general activities such as feeding, and subsequently on overall health [69]. There is an argument that technological systems do not need to monitor ADL at the same level of detail that current ADL tests provide, and that in some cases the level of intrusion required to monitor a sub-activity of the ADL is unfeasible. For example, the Barthel Index ADL of “grooming” specifies activities such as teeth brushing, whereas current technology rarely recognises specific grooming activities. This may be due to privacy issues surrounding the placement of technology in bathrooms [28], creating a reliance on technology to indicate an individuals’ presence in the room but not the specific activities occurring inside. One system was able to recognise tooth brushing [42]; however, this system consisted of only wearable sensors that several studies have noted are not currently well accepted by older adults [20,23,41]. Therefore, the inclusion of multiple sub-activities may necessitate further investigation of the hierarchical or binary nature of individual sub-activities in determining whether the global task, such as feeding, has been achieved but also whether some data or sub-activities are redundant measures in the decision-making process.

Some of the ADL recognised are not associated with common assessments of independent living ability, such as the Barthel ADL index or Lawton and Brody iADL scale, namely, “sleep” and “social interaction”. Both sleep and social interaction may have a large impact on functional ability of other ADL; however, this interaction is currently not well understood or included within objective, technology-based measures. There is a growing appreciation of the need for a biopsychosocial approach to healthcare [72], which places a greater importance on social interaction and the role this plays in overall wellbeing, hence the need to include “social interaction” as an important ADL action in monitoring technology. As the systems can continuously monitor activity, they are able to monitor these ADL without increasing the burden on caregivers, which may help to explain why they are so commonly included.

The general nature of the “feeding” and “grooming” ADL can explain why they are recognised by most of the systems identified, whereas the more specific ADL of “stair usage” and “medicine” are recognised much less frequently (Figure 2). “Stair usage” was only recognised by one system [42], despite it being one of the key activities required to assess independent living and one of the main activities older adults tend to struggle with [73]. Given the high injury, and potential mortality risk associated with stairway falls [73], it seems strange that this activity is rarely recognised and should be considered with more importance in future systems.

### 4.2. Sensor Types and Combinations

Since 2014, ADL recognition systems have become more complex in terms of the range of sensor types included; however, this has not correlated to a wider range of recognised ADL. Many of the developments in recent years have related to identifying ADL that are difficult to recognise due to privacy issues; for example, introducing humidity and sound sensors to detect bathing activity. Over time, there has been a tendency away from sensors requiring greater user interaction, such as RFID tags or wearable sensors, which may reflect the desire of older adults to have sensors that they are not necessarily aware of. The reasons for the lack of interest in these sensor types should be investigated further, as they may present a reasonable solution to current issues within monitoring systems, such as the ability to distinguish separate people in a cohabitation setting.

Most current systems favour motion sensors (Figure 4), as they can detect movements within certain areas, and suggest the level of activity of an individual through calculating the number of movement events detected within a certain time [39]. This ability means that a wide range of ADL can be recognised through inference of time spent in a specific room or area of a living space; for instance, time spent in a kitchen can imply meal preparation activity. However, just because someone is present in a room, does not mean that the expected activity is occurring [28], and therefore motion sensors alone may not be the most appropriate method of ADL recognition despite their high level of acceptance among older adults [43]. As a result, several systems use a combination of technology types (Table 4), using motion sensors to identify room activity and other technology such as wearable sensors [39] or power consumption sensors [37,66] to recognise the specific activity occurring within the room.

Environmental-only systems were the most common in the current survey, as they can improve the accuracy of activity recognition [54,57] without relying on direct participant interaction through wearable sensors [20]. Most combination systems use two sensor types [28,37,41,43,45,48,52,64,66], for example, motion sensors combined with power sensors, to provide complementary information related to an activity and improve the recognition accuracy [57]. Arguably, a wider range of sensors would allow the most accurate activity recognition as several aspects of the activity could be recognised at once. For example, the seven-sensor-type system used by [60] can accurately recognise the sub-activity of bathing (part of the “grooming” ADL, Table 4) as the door sensor would indicate entrance into the bathroom, the acoustic sensor recognises the sound of water with humidity sensors also indicating the presence of water usage, and power sensors can indicate the use of an electric shower. However, there are concerns surrounding the cost of installing and maintaining complex systems [41] and the difficulty in handling the high volume of data they generate [51,74]. This may explain the preference of commercial systems to use simpler configurations of two-sensor types [41,45,46,48] or three-sensor types [47,50].

Another issue with the current monitoring systems is the ability to distinguish the ADL function of different people within the same space, and therefore many are not suitable for multi-occupancy housing [39]. To overcome this, a system was developed combining a wearable accelerometer with passive infrared motion sensors [39], which can communicate and therefore identify an individual. Although this appears to be a logical solution, it is still reliant on the individual wearing a sensor, which can lead to issues relating to individual compliance [20]. Although wearables are arguably able to provide more precise data relating to body movements and postural changes [19], and may be linked to fewer privacy concerns than image-based sensors [69], they are considered too intrusive by many older adults [20,23,41].

### 4.3. ADL Recognition Method

Several of the systems recognised the ADL indirectly based on a sequence of interactions with the environment (Table 5), which suggest a certain activity has occurred [25]. For example, “feeding” can be inferred from a combination of room sensors in the kitchen, door sensors on a fridge, and power sensors attached to a microwave. For the current aims of these systems, which involve recognising a potential decline within an ADL action rather than directly assessing the performance of an ADL action [21], directly measuring movement patterns is not necessary, which may explain the preference for indirect recognition methods. However, as the population continues to age, directly assessing ADL performance may become more useful as it will highlight exactly where an individual is having difficulty without the reliance on traditional tests, which can be time consuming. This will reduce some of the burden on caregivers and allow more time to be dedicated to designing and implementing interventions. As these interventions will be based on objective performance data rather than subjective measures, healthcare efficiency may be improved. Healthcare professionals should be consulted on this process, as it has been argued that introducing technology does not always improve efficiency due to the required changes in habitual working practices [41].

### 4.4. Limitations and Future Directions

There were some limitations within this review. Firstly, it aimed to identify the types of technology currently used within recognition systems, and therefore did not include the specific number of sensors used within each system. Future work should identify the most effective set-ups within systems, in terms of the number of sensors included, and where they are placed within the living environment. The scope of this review was also limited in that only ubiquitous systems consisting of wearable and/or environmental systems were included. This means that other technological advances, such as systems including artificial intelligence, or the role of social robots, were not considered despite their potential in supporting ageing-in-place. Although social robotics and AI share the same sensor technology as those reviewed in this article, they provide a crucial, separate contribution in terms of data analysis (AI) and novel forms of interfacing (robotics), whereby the user is prompted to perform certain behaviours. These aspects of monitoring technology were beyond the scope of this review; however, they should be the focus of future work.

Future work should establish a clear consensus as to the key ADL required from an older adult and healthcare worker perspective, the most appropriate sensor types to utilise, and the most suitable method of ADL recognition to use in order to enhance the effectiveness of current ADL monitoring systems [74]. This may include an element of routine monitoring alongside specific ADL recognition, as deviations from the routine can highlight changes in cognitive function often missed by the traditional measures, and therefore could provide a greater insight into the individual’s functional decline [39,43]. The involvement of key stakeholders, such as older adults and healthcare workers, should be prevalent within any future work [75,76,77] to ensure that the systems are both functional and usable; there is little point in developing a system capable of highly detailed ADL recognition if those it is designed to help are unable or unwilling to use it. This will help to ensure that any future developments are accepted by those whom it is intended to help, and that advancements are being made because they are needed, not simply because they can be.

## 5. Conclusions

This scoping review aimed to identify the systems currently used to recognise the ADL of community-dwelling older adults. This included discerning the ADL recognised, the types of technology used, and the method employed for ADL recognition.

Fourteen ADL were recognised, ranging from basic ADL, including feeding and grooming, to more complex iADL, such as medicine management and social ADL, including time spent away from home and the presence of visitors. However, there was some disagreement surrounding the specific activities that were included within the ADL of “feeding”, “grooming”, and “social interaction”. There is a need to come to a consensus on the most important ADL, and the level of specificity relating to ADL recognition required from these systems.

In terms of the types of technology used, environmental-only systems were the most common, with wearable sensors rarely used. Door and motion sensors are the most common technology types, used on their own or in combination with other sensors such as power consumption, light, or temperature. Systems ranged in complexity, from utilising one to seven types of technology, although this did not appear to correlate with an increase in the number of different ADL recognised. Therefore, more work is needed to understand the most efficient combinations of technology types in terms of both functionality and user acceptability.

Most ADL were inferred from room activity rather than direct measurement, implying that systems are limited in terms of their ability to detect detail relating to ADL performance. Currently, the systems can identify when changes in ADL performance occur but are unable to determine why. This restricts their usefulness as a tool for aiding healthcare decisions, as there is still a need for further tests or subjective measures to determine the exact cause of a decline in function. Through enabling a more detailed tracking of ADL function, we will be able to better understand the disablement process and therefore develop more efficient interventions.

## Figures and Tables

**Table 1:** Inclusion and exclusion criteria for the paper selection process.

Inclusion	Exclusion
- Uses ADL function as outcome measure - Uses named sensors to recognise ADL functioning - Independent older adults being monitored in their home, or “free-living” environments	- Focus on “fall risk” or “fall detection” - Focus on “physical activity monitoring” - Focus on “assistive technology” - Lab-based only trials - Papers published before 2008

**Table 2:** Search terms used to identify the appropriate published research.

Property Name	Keywords
Population	ag* OR elder* OR older
	AND
	“free living” OR “community dwelling” OR home
Activity	“activities of daily living” OR ADL
	AND
Measurement	monitor OR assess* OR detect OR measur* OR recogni*
	AND
Technology	techn* OR wearable OR sens* OR device OR app OR smartphone OR “smart home” OR “human activity recognition” OR HAR

(*) has been included after certain word stems, such as ‘ag’, to broaden the search and allow any word variation to be included (e.g. ‘ageing’, ‘aged’) without having to include each one as a separate search term.

**Table 3:** Definitions of the Activities of Daily Living (ADL) recognised by the identified systems.

Activity	Definition
Bed Usage	Time spent in bed, including movements while in bed which can infer sleep quality [26]
Dressing	Activity performed in standing posture, in the bedroom or bathroom immediately following getting out of bed or following a visit to the toilet [39]
Feeding	Succession of movements performed in a kitchen environment during a reasonable duration and at selected moments in the day, including eating and meal preparation activity [39]
Grooming	Activity relating to maintaining personal hygiene (showering, shaving, brushing teeth & styling) [20,40]
Household	Any activity relating to household chores, for example laundry or washing up [11]
Medicine	Able to manage medication [11]
Mobility	Number of activated sensors and total distance covered walking inside the apartment per day [10]
Recreation	Sitting at a table or easy chair [20,40]
Sleep	Resting at night or napping either in bed or on the couch [40]
Social Interaction	Time spent away from the property [41], or the detection of visitors within the home [19]
Stair Usage	Walking up or down stairs without falling/tripping [42]
Toileting	Spending less than 5 min in a bathroom [23]
Transferring	The ability to move oneself from/to a bed or a chair [39]
TV Usage	Television is on, usually with a main focus on the TV but can include doing other activities while the television is on [40]

**Table 5:** Method used to recognise each ADL action, where direct refers to specific activities that cannot be mistaken for something else, and indirect refers to inferred recognition based on a “most likely” scenario.

Activity	Direct/Indirect/Both	Outcome Measure
Bed Usage	Direct	Pressure sensors or accelerometers attached to the bed
Dressing	Indirect	Room activity and door sensors attached to specific drawers/wardrobes
Feeding	Both	Room activity, appliance use, or door sensors attached to cupboards within the kitchen area (inferred activity) Wearable sensor data (direct measure)
Grooming	Indirect	Room activity, changes in temperature/humidity, water usage or specific door usage
Household	Indirect	Water usage, room activity or appliance usage
Medicine	Indirect	Door sensors attached to medicine cabinets
Mobility	Both	Room activity (inferred activity) Wearable sensors (direct measure)
Recreation	Indirect	Room activity or power consumption
Sleep	Indirect	Room presence (but inactivity), typically the living room & bedroom
Social Interaction	Indirect	Door sensors attached to the main property entrance
Stair Usage	Indirect	Combined wearable sensors
Toileting	Indirect	Room activity, specific door usage, water usage or accelerometers attached to the flush mechanism
Transferring	Both	Room activity or pressure sensors (indirect) Wearable sensors (direct measure)
TV Usage	Direct	Power consumption/smart switch

## Data Availability

No new data were created or analyzed in this study. Data sharing is not applicable to this article.

## Supplementary Materials

The following are available online at https://www.mdpi.com/1660-4601/18/1/163/s1, Table S1: Details of each systems included within this review. The reference values shown here are referred to throughout the manuscript and relate to the corresponding value in the reference list.
