# Supplementary material for: Technology Used to Recognize Activities of Daily Living in Community-Dwelling Older Adults
Source: Int J Environ Res Public Health. 2020 Dec 28;18(1):163. doi: 10.3390/ijerph18010163 (PMC7795436; doi:10.3390/ijerph18010163)
Supplement: Supplementary file 1 [file ijerph-18-00163-s001.pdf]

Supplementary Table S1) Details of each systems included within this review. The reference values shown here are referred to throughout the manuscript and relate to the corresponding value in the reference list.

Reference	Year	System / Sensor Name	Recognised ADL	Type of Sensor/s included	Outcome Measure
Commercial ADL recognition systems not used in research					
[44]	/	MiMonitor	Feeding, Grooming, Mobility, Social interaction	Door contact	Door usage
				Motion	Room movement
				Video Camera	
[45]	2004	Just Checking	Grooming, Mobility, Social interaction	Door contact	Door usage
				Motion	Room movement
[46]	2013	Canary Care	Grooming, Mobility, Social interaction	Door contact	Door usage
				Motion	Room movement
				Light	Room usage
				Temperature	
[47]	2015	Memo-Hub	Feeding, Grooming, Mobility, Social interaction, TV watching	Door	Door usage
				Motion	Room movement
				Power consumption	Electrical appliance use
[48]	2017	Tec-Angel	Grooming, Mobility, Social interaction	Door contact	Door usage
				Motion	Room movement
[49]	2018	Text Care	Feeding, Grooming, Mobility, Social interaction	Door contact	Door usage
				Light	Room usage
				Sound	
				Temperature	
				Motion	Room movement
[50]	2019	Howz		Door contact	Door usage

			Feeding, Grooming, Mobility, Social interaction, TV usage	Motion	Room movement
				Power consumption	Electrical appliance use
Commercial ADL recognition systems also used in research					
[19]	2019	Canary Care (2013) / /	Feeding, Grooming, Sleep, Social interaction, Toileting	Door contact	Door usage
				Motion	Room movement
				Light	Room usage
				Temperature	
				Grid-eye	Object interaction
				Power consumption	Electrical appliance use
[41]	2019	LivOn	Feeding, Grooming, Mobility, Sleep, Social interaction, Toileting	Door contact	Door usage
				Motion	Room movement
Named sensors used in research (but not as commercially available systems for the purpose of ADL recognition)					
[51]	2011	WPC15.4	Feeding, Grooming, Household, Toileting	Power consumption	Electrical appliance use
[24]	2012	Eventlogger	Bed usage, Dressing, Feeding, Grooming, Household, Recreation, Toileting, TV Usage	RFID Tag	Object interaction
[52]	2013	Flexiforce sensor	Bed usage, Feeding, Grooming, Recreation, Toileting, TV watching	Force/pressure sensor	Applied force
		/		Power consumption	Electrical appliance use
[53]	2014	Kinect	Feeding, Mobility, TV usage	Depth Camera	Room movement Object interaction
[54]	2015		Feeding, Household, Mobility, Recreation		

[20]<br>[40]	2014<br>2015	SHT21P	Bed usage, Feeding, Grooming, Recreation, Sleep, Toileting, TV usage	Humidity	Room usage
		DS18B20		Temperature	
		AMS302		Light	
		EKMB1101111		Motion	Room movement
		ADXL345		Accelerometer	Bed/chair movement<br>Toilet flush movement
[42]	2015	MSP430CPU	Feeding, Grooming, Household, Mobility, Recreation, Sleep, Stair use, TV usage	Accelerometer	Wrist orientation /movement
				Altimeter	
				Temperature	
		Gadgeteer FEZ Cerberus		Barometer	
				Gyroscope	
				Light	
[28]	2016	/	Grooming, Household, Mobility, Toileting	Hydro	Water pressure
		MS 16A		Motion	Room movement
[23]	2016	ADNS-3060	Bed usage, Feeding, Grooming, Recreation, Sleep, Social interaction, Toileting, TV usage	Video Camera	Video Footage
[43]	2018	DS90	Mobility, Sleep, Social interaction, Toilet use	Door contact	Door usage
		MS13E		Motion	Room movement
[69]	2019	Pandlets	Feeding	Accelerometer	Wrist orientation/movement
				Gyroscope	
[67]	2020	Everspring HSP02		Motion	Room Movement

		Everspring HSM02	Feeding, Grooming, Recreation, Sleep, Social interaction	Door Contact	Door Usage
		Aeotec ZW078 & ZW096		Power Consumption	Electrical Appliance Use
[68]	2020	Panasonic EKMB1101112	Feeding, Grooming, Sleep, Social interaction	Motion	Room Movement
		Omron 2JCIR-BL		Humidity	Room Usage
				Light	
				Barometer	
				Sound	
				Temperature	
		Rohm STM250J		Door Sensor	Door Use
Unnamed sensors used in research (but not as commercially available systems for the purpose of ADL recognition)					
[55]	2008	/	Bed usage, Dressing, Feeding, Grooming, Toileting, Transferring	Motion	Room movement
[56]	2008	/	Feeding, Grooming, Recreation, Sleep, Toileting	Motion	Room movement
[57]	2010	/	Feeding, Grooming, Recreation, Sleep, Social interaction, TV usage	Motion	Room Activity
[26]	2011	/	Bed usage, Feeding, Grooming, Medicine, Recreation, Sleeping, Social interaction, Transferring	Accelerometer	Bed movement
				Door contact	Door use
				Humidity	Room usage
				Light	
				Motion	Room movement
[39]	2012	/	Dressing, Feeding, Mobility, Recreation, Sleep, Toileting	Motion	Room movement
				Wearable Accelerometer	Postural changes

[37]	2012	/	Feeding, Grooming, Sleeping, Tv usage	Motion	Room movement
				Power consumption	Electrical appliance use
[25]	2014	/	Dressing, Feeding, Grooming	Door contact	Door usage
				Motion	Room movement
				Power consumption	Electrical appliance use
				Temperature	Room usage
[58]	2014	/	Grooming, Toileting	Motion	Room movement
[59]	2014	/	Feeding, Grooming, Recreation, Sleeping, Toileting	Force/Pressure	Sitting/lying
				Motion	Room movement
				Power consumption	Electric appliance use
				Temperature	Room usage
[60]	2014	/	Dressing, Feeding, Grooming, Mobility, Transferring	Accelerometer	Bed/chair movement
				Sound	Water usage
				Humidity	Room usage
				Temperature	
				Motion	Room movement
				Power consumption	Electrical appliance use
				Door contact	Door use
[61]	2015	/	Bed usage, Feeding, Grooming, Household, Medicine, Recreation, Sleep, Social interaction, Transferring	Door contact	Door use
[62]	2015	/	Bed usage, Dressing, Feeding, Grooming, Mobility, Sleep	Accelerometer	Bed movement
				Motion	Room movement
				Power consumption	Electrical appliance use
				Door contact	Door usage
				Temperature	Room usage

				Humidity	
[63]	2016	/	Feeding, Grooming, Recreation, Sleep, Social interaction, Transferring	Door contact	Door usage
				Motion	Room activity
				Light	Room usage
				Temperature	
[64]	2017	/	Feeding, Recreation, Sleep, Social interaction	Door	Door usage
				Motion	Room movement
[10]	2018	/	Feeding, Grooming, Mobility, Recreation, Sleep, Social interaction, Toileting	Motion	Room movement
[65]	2018	/	Feeding, Grooming, Recreation, Sleep, Toileting, TV watching	Force/Pressure	Sitting/Lying
				Motion	Room activity
				Power consumption	Electrical appliance use
				Temperature	Room usage
[66]	2019	/	Feeding, Grooming, Household, Recreation, Sleep, Social interaction	Motion	Room movement
				Power consumption	Electrical appliance use
